# Experimental evolution reveals high insecticide tolerance in *Daphnia* inhabiting farmland ponds

**DOI:** 10.1111/eva.12253

**Published:** 2015-03-21

**Authors:** Mieke Jansen, Anja Coors, Joost Vanoverbeke, Melissa Schepens, Pim De Voogt, Karel A C De Schamphelaere, Luc De Meester

**Affiliations:** 1 Laboratory of Aquatic Ecology, Evolution and Conservation, KU LeuvenLeuven, Belgium; 2 ECT Oekotoxikologie GmbHFlörsheim a.M, Germany; 3 Biodiversity and Climate Research Centre (BiK-F)Frankfurt a.M, Germany; 4 Institute for Biodiversity and Ecosystem Dynamics (IBED), Universiteit AmsterdamAmsterdam, The Netherlands; 5 Laboratory for Environmental Toxicology and Aquatic Ecology, Environmental Toxicology Unit (GhEnToxLab), Ghent UniversityGhent, Belgium

**Keywords:** adaptation, carbaryl, *Daphnia magna*, evolutionary potential, insecticide tolerance

## Abstract

Exposure of nontarget populations to agricultural chemicals is an important aspect of global change. We quantified the capacity of natural *Daphnia magna* populations to locally adapt to insecticide exposure through a selection experiment involving carbaryl exposure and a control. Carbaryl tolerance after selection under carbaryl exposure did not increase significantly compared to the tolerance of the original field populations. However, there was evolution of a decreased tolerance in the control experimental populations compared to the original field populations. The magnitude of this decrease was positively correlated with land use intensity in the neighbourhood of the ponds from which the original populations were sampled. The genetic change in carbaryl tolerance in the control rather than in the carbaryl treatment suggests widespread selection for insecticide tolerance in the field associated with land use intensity and suggests that this evolution comes at a cost. Our data suggest a strong impact of current agricultural land use on nontarget natural *Daphnia* populations.

## Introduction

Pesticides are widely used in agriculture to increase crop yield (Waterfield and Ziberman 2012). Spray drift or run-off of pesticides can affect natural, nontarget populations inhabiting surface waters situated in the vicinity of the treated land area, impacting community structure and ecosystem functioning (Parker et al. 1999). Several studies have shown that exposure to toxic substances may induce micro-evolutionary responses in natural populations, leading to genetic differences in the concentration–response curve between exposed and nonexposed populations (e.g. Raymond et al. 2001; Medina et al. 2007; Jansen et al. 2011). Genetic adaptation to pesticides may also come at a cost, however, such as an increased susceptibility to parasites (Jansen et al. 2011) and loss of genetic diversity (Coors et al. 2009).

The water flea *Daphnia* (Crustacea) belongs to the freshwater zooplankton and is a key model organism in ecology and evolution (Lampert 2006; Decaestecker et al. 2007; Van Doorslaer et al. 2009a; Colbourne et al. 2011; Jansen et al. 2011; Miner et al. 2012) and in ecotoxicology (OECD 2004; Walker et al. 2006). Their cyclic parthenogenetic reproduction allows the exposure of single genotypes to a range of environmental conditions using clonal replicates, to quantify genotype-dependent responses to environmental gradients. The short generation time allows monitoring responses in experimental evolution trials (Van Doorslaer et al. 2009b; Jansen et al. 2011), while the production of dormant stages (ephippia, Hebert 1978) that accumulate in dormant egg banks allows obtaining representative samples of the gene pool of natural populations. Large-bodied *Daphnia* species such as *D. magna* play a central role in the food web of eutrophic ponds and shallow lakes. Such habitats are often quite abundant in agriculture landscapes, where exposure of *Daphnia* populations to pesticides through drainage, spray drift or run-off may be common.

The aim of this study was to investigate whether natural *Daphnia magna* populations inhabiting ponds in agricultural landscapes possess evolutionary potential for adaptation to changes in the level of pesticide exposure, using carbaryl as a model acetylcholinesterase-inhibiting insecticide. To that end, we carried out a selection experiment starting from standing genetic variation sampled by hatching dormant eggs from the egg bank of seven natural populations. After the selection experiment, we performed acute toxicity tests to compare the carbaryl tolerance of the experimental populations that had been exposed to high concentration pulses of carbaryl, the experimental populations that had been exposed to control conditions, and the original field populations. We tested two hypotheses. First, we tested the hypothesis that natural populations of this nontarget species harbour evolutionary potential to respond to selection exerted by pulse exposure to carbaryl. Second, we tested the hypothesis that the selection background of the natural populations (i.e. the selection conditions in their original habitat) influences their response to insecticide selection.

## Materials and methods

Our overall research strategy involved a selection experiment on carbaryl tolerance in outdoor containers followed by an assessment of the genetic changes in tolerance to carbaryl in laboratory short-term exposure experiments.

### Daphnia populations

We exposed dormant eggs contained in the sediments of seven ponds and small shallow lakes in Flanders known to be inhabited by *D. magna* (Blankaart, Uitkerke, Moorsel, OM 1, OM 2, Oud-Heverlee and Tersaart; see Coors et al. 2009) to favourable hatching stimuli (i.e. exposure to ‘spring conditions’: fresh noncontaminated dechlorated tap water, long day photoperiod 16L/8D and 20°C, after storage of the eggs for > 1 month at 4°C in the dark; see De Meester and De Jager 1993), and randomly isolated 125 hatchlings from each population. Dormant eggs in *D. magna* are produced sexually, so all hatchlings are genetically unique. These hatchlings were cultured in isolation to establish clonal lineages. The seven study populations here are a subset of the populations studied by Coors et al. (2009) in their analysis of the association between carbaryl tolerance of 10 *D. magna* populations and land use intensity in the immediate neighbourhood of their habitats.

### Selection factor carbaryl

The pesticide carbaryl (1-naphthyl methylcarbamate, CAS 63-25-2, purity 99.8%, Sigma-Aldrich, Germany) belongs to the group of carbamates, which together with the organophosphorous insecticides act as acetylcholinesterase inhibitors. Carbaryl prevents the breakdown of the chemical messenger acetylcholine between the synapses in the nervous system. This results in an overload of acetylcholine in the synaptic cleft leading to overstimulation of the postsynaptic receptors. The receptors are then no longer able to contract or relax in response to a synaptic stimulus (Walker et al. 2006). Since 2007, carbaryl has no marketing authorisation as agricultural insecticide in Belgium anymore, but already sold products may still be used and the VMM (Vlaamse Milieu Maatschappij) still reports carbaryl concentrations in the field (Vlaamse Milieu Maatschappij 2010).

### Experimental evolution trials

Using the hatchlings obtained from the sampled dormant egg bank of each of the seven natural populations, we carried out a selection experiment in outdoor containers. We applied two treatments: a carbaryl exposure treatment and an ethanol control treatment, with two replicate containers for every population x treatment combination, giving 28 experimental units. We used 28, 225 L containers filled with 180 L of dechlorinated tap water and covered by mosquito netting to keep away predatory midge larvae. Two weeks before the inoculation of the *D. magna* juveniles, we added an inoculum of 6 × 10^8^ cells of the unicellular green alga *Scenedesmus obliquus* to each container (i.e. 3.3 × 10^3^ cells mL^−1^). The growth of these algae during the 2 weeks before inoculation of the *Daphnia* provided sufficient food for rapid population growth of the *Daphnia* upon their inoculation. The alga community was not replenished during the experiment and was self-maintained.

In each container, we inoculated clonal descendants of the 125 genetically unique hatchlings of a given *D. magna* population. The inoculate used can be considered a representative sample of the standing genetic variation present in the natural population at the start of a growing season, when populations in nature hatch from their dormant egg banks. Each container received one individual (< 5 days old) from each of 125 clones derived from a single population, resulting in a start density of 0.70 individuals per litre; the inoculated individuals were first-generation and second-clutch offspring of the animals that hatched from the dormant egg bank. For each population, all containers (two replicates × two treatments) received the same, standardized set of unique clones. Carbaryl exposure was achieved by exposing the populations to three pulses of carbaryl (nominal concentration of 32 *μ*g L^−1^) that were given at an interval of 14 days to mimic spray season, starting 2 weeks after stocking the containers with *D. magna* juveniles. Exposure to 32 *μ*g L^−1^ can be considered a strong selection pressure, as the EC_50_ value for carbaryl for those populations has been estimated to be around 8 *μ*g L^−1^ for juveniles (Coors et al. 2009). At the same time as the carbaryl pulses, ethanol pulses were given in the control treatments, exposing the animals to the same concentration of ethanol used as carrier solvent as in the carbaryl exposure treatment (1 mL ethanol (pure, 99%) per pulse and per container i.e. 0.004 mL L^−1^).

The container selection experiment ran in total for 80 days, with 55 days after the first pulse of carbaryl, representing approximately 5–8 generations; 27 days after the last pulse was given, the selection experiment ended.

### Clones used for the acute toxicity tests

At the end of the selection experiment, we randomly isolated 10 individuals from each population × treatment combination (i.e. pooled over both replicate containers) to start clonal lineages in the laboratory. Random extinction of clones after isolation reduced the number of available lineages to 3–6 per population × treatment combination (see Table S1). These losses of clones were caused by the prolonged period (approx. 82 generations) of culture under suboptimal stock conditions following isolation of the lineages. During this period, the cultures were only fed twice a week. Extinction of clones was random, that is independent of population of origin nor selection history (verified by one-way anova on number of clones that survived: population: *F* = 0.547, df = 6, 14 and *P* = 0.77; selection history: *F* = 1.98, df = 2, 18 and *P* = 0.17).

After the selection experiment, we obtained three sets of clonal cultures for each of the seven sampled natural populations: (i) a set of genotypes directly hatched from the dormant egg banks (called original populations; they are a subset of the lineages that were used to inoculate the containers) and never exposed to selection in the containers, (ii) a set of genotypes obtained from the control treatment in the container experiment (control populations), and (iii) a set of genotypes obtained from the outdoor containers exposed to carbaryl (carbaryl-selected population). Of the 102 lineages tested for their sensitivity to carbaryl, the 33 clonal lineages directly hatched from the original populations are genetically unique as they were hatched from dormant eggs, which are the result of sexual recombination. The remaining 69 clonal lineages were isolated from populations that were reproducing parthenogenetically, so that in principle several isolates may belong to the same clone; of these, 34 were screened for their variation at seven polymorphic microsatellite markers (Jansen et al. 2011) and proved to be unique clones. Unfortunately, the remaining 35 clones could not be genotyped because they were accidently lost before we could genotype them using microsatellites. The fact that all 34 genotyped clones proved genetically unique, however, suggests that the likelihood that our results would be influenced by different isolates belonging to the same genotype is small.

### Acute toxicity tests

We carried out standard acute toxicity tests for a total of 102 clonal lineages (*n* = 4–6 lineages per population, for two populations *n* = 3; Table S1) based on the OECD 202 guideline acute *Daphnia* test (Organisation for Economic Co-Operation and Development, OECD 2004).

For two generations prior to the start of the experiment, all isolates were grown under standardized conditions in terms of density and medium to minimize maternal effects. For each clonal lineage, four to six cultures were maintained to generate enough juveniles for the toxicity experiments. In each culture, ten individuals are raised as a cohort in 500-mL jars filled with Aachener Daphnien medium (ADaM, Klüttgen et al. 1994) in a climate-controlled room with a constant temperature (20 ± 1°C) and a fixed day/night regime (16 h light/8 h dark), and daily fed 1 × 10^5^ cells mL^−1^ of the unicellular green alga *Scenedesmus obliquus* until maturation, after which food levels were doubled.

For the acute toxicity tests, we exposed second or third clutch neonates < 24 h old to eight different carbaryl concentrations ranging from 4 to 25.1 *μ*g L^−1^ (spacing factor of 1.3 between concentrations) and an ethanol control.

The analysis of the actual concentrations of carbaryl in test solutions was carried out according to the method described by Cerbin et al. (2010). Briefly, water samples for chemical analyses of carbaryl were preserved with formic acid until analysis. A LC–MS/MS [4000QTrap (Applied Biosystems, Gent, Belgium) operated with electrospray ionization in the positive ion mode (ESI)] was used for the determination of the actual concentrations of the pesticide. The separation was done on a C18 column [Pathfinder (Shimadzu, Duisburg, Germany): 4.6 × 50 mm, silica 300, particle size 3.5 *μ*m], using a mobile phase consisting of methanol and water, both with 5 mm of NH_4_Ac. The transitions 202→145 and 202→127 were monitored.

For each clonal lineage and test concentration, four cohorts of five neonates randomly collected from the juveniles produced in the 500-mL cultures were inoculated in 5 mL ADaM in a 30-mL glass jar. All juveniles were first pooled after which we randomly picked out five individuals for each exposure unit (see guideline OECD 202: OECD 2004). Stock solutions of carbaryl were prepared at eight concentrations in ethanol and stored at −20°C.

Given the size of the experiment, with parallel full scale standardized acute toxicity tests on 102 clonal lineages, tests were split over 86 different days. The order in which clonal lineages were tested was randomized across populations. We minimized the time between inoculating the experimental animals and the carbaryl pulse and ensured that all animals tested on 1 day were exposed to their test concentration at the same moment. Each test included a solvent control with 0.005% ethanol, the same ethanol concentration as in all carbaryl test solutions. At the start of the experiment, we also included a blank control containing only ADaM, but as there was no difference (between 0 and 10%) in mortality in both controls (blank and ethanol control), blank controls were not included in further exposure assays. In case there was more than 10% mortality in the control treatment, we decided to remove that specific test from the analysis according the OECD guideline 202. Experimental animals were not fed during the acute toxicity test and tested in a dark/light cycle (16 h light/8 h dark). After 48 h, all individuals were scored for immobility. On each test day, four different variables were measured in the exposure medium: pH, conductivity (*μ*S cm^−1^), oxygen level and temperature (°C) (Table S2).

### Statistical analysis

#### Concentration–response curves and response to selection

Generalized linear modelling (GLiM) was applied to analyse acute toxicity response data (proportion immobilized daphnids after 48 h exposure versus log_10_-transformed carbaryl concentrations) and to estimate the median effect concentration (EC_50_) for each population × treatment combination. We used GLiM with a probit link and assumed binomial error distribution as recommended by McCullagh and Nelder (1989) in the statistical software R (R Development Core Team, 2007). To account for extrabinomial variation (i.e. overdispersion expressed as the residual deviance being more than 1.5 greater than the degrees of freedom of the residual deviance), we used as model family ‘quasibinomial’ and replaced *χ*² tests by *F*-tests as recommended by Crawley (2007). The results of the quasibinomial model differ from the binomial model only with regard to the standard errors of the parameter estimates, with the quasibinomial model yielding larger and therefore more conservative confidence intervals for point estimates such as the EC_50_. The 95% confidence intervals of the EC_50_ were calculated from the variances and covariances of the model parameters using Fieller's theorem as outlined by Kerr and Meador (1996) and Wheeler et al. (2006).

As the accuracy of the estimate of the EC_50_ is greater when replicate observations are combined in one analysis (OECD 2004), we fitted one concentration–response curve for each population × treatment combination (original, control and carbaryl-selected for each of the seven populations) using the results of all replicate trials with the different clones of this population. A backward elimination process (Crawley 2007) was applied to analyse the influence of origin of the populations (factor ‘population’) and carbaryl exposure during the selection experiment (factor ‘selection history’) with regard to the toxicity of carbaryl on the clonal lineages. Starting with the most complex model explaining the proportion of dead daphnids (y∼ carbaryl × population × selection history, i.e. taking all possible interactions into account), the term that caused the least reduction in deviance was deleted in each step. This stepwise reduction process was applied until the least complex model was identified that significantly reduced deviance in comparison with the next more complex model. Following the analysis on all populations, we contrasted selection histories to analyse specifically the influence of differential selection due to carbaryl exposure within the experiment (by comparing ‘control’ and ‘carbaryl-selected’ populations) of the overall selection experienced by the carbaryl-selected populations (by comparing only ‘original’ and ‘carbaryl-selected’ populations) and of the ‘background’ selection caused by the containers (by including only ‘original’ and ‘control’ populations). In addition to this GLiM analysis, we also carried out a general linear model (anova) on the EC_50_ values calculated from concentration–response curves estimated for each clonal isolate of the different treatments and populations separately, with population and selection history as independent variables (see Table S3; run using Statistica v12, Statsoft 2014: http://www.statsoft.com). This anova approach uses the EC_50_ values estimated for each clone separately, and while it incorporates variation among clones for this variable, it has the disadvantage compared to the GLiM analysis that the single clonal EC_50_ values are less informative than considering the whole concentration–response curve per population because they only represent one point estimate of the response and are calculated from a smaller data base (i.e. on the four replicate cohorts of five individuals tested per clone and concentration).

#### Relationship with land use

To test for the hypothesis that the response to selection is dependent on the selection background of the study populations, that is the degree to which they are impacted by intensive agriculture, we quantified the relationship between the relative change in carbaryl tolerance during the selection experiment and the land use intensity in the immediate vicinity of the habitats the populations were derived from. The relative change in carbaryl tolerance during the selection experiment was described by the log_2_ of the ratio of the EC_50_ of each container population (‘control’ or ‘carbaryl-selected’) over the EC_50_ in the respective original population [log_2_ (EC_50_ treatment/EC_50_ original)]. This was calculated for both control and carbaryl-selected populations separately. A value above 0 indicates an increase in tolerance in comparison with the original population and a value below 0 a loss in tolerance, respectively. Using this ratio of EC_50_ values, the change in carbaryl tolerance was analysed by Spearman rank correlation for its association with land use intensity in the vicinity of the pond of origin of the populations. In earlier work, we documented that carbaryl tolerance of *Daphnia* populations tends to increase with increasing intensity of land use in the immediate vicinity of their habitat (Coors et al. 2009). The seven ponds from which the experimental populations were selected were ranked for land use intensity (1 = lowest and 7 = highest land use intensity) based on the visual screening of the percentage of crop (corn and cereals), pasture and garden area in a radius of 50 m around the pond, and using the percentage of cropland as the main criterion (as described in Coors et al. 2009; see Table S4). This assessment was based on earlier work that established that water quality in farmland ponds is largely determined by land use nearby (< 200 m) the ponds (Declerck et al. 2006).

## Results

### Concentration–response curves and response to selection

**Figure 1:**
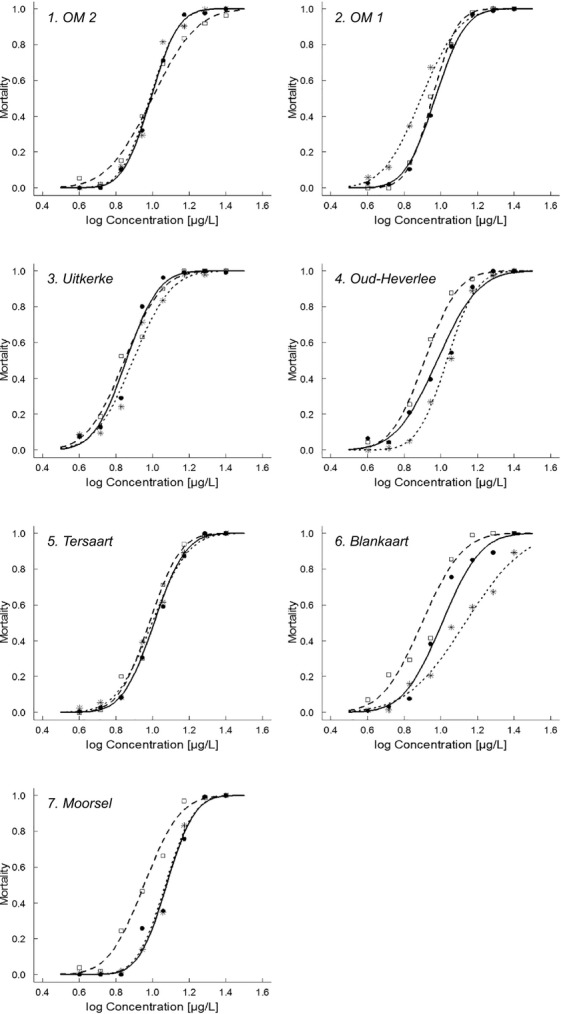
Concentration-response curves for carbaryl for the study populations as sampled from the dormant egg banks (original field populations, full line and circles), the control populations (control condition in experimental evolution trial; dashed line and squares) and the carbaryl-selected populations (from the experimental evolution trial; dotted line and stars) for each of seven natural *Daphnia magna* populations isolated from Flemish farmland ponds situated in areas that differ in land use intensity. The figures of the different populations are ranked based on land use intensity in the neighbourhood of the pond (OM2: low land use – Moorsel: high land use; see Table S4).

**Figure 2:**
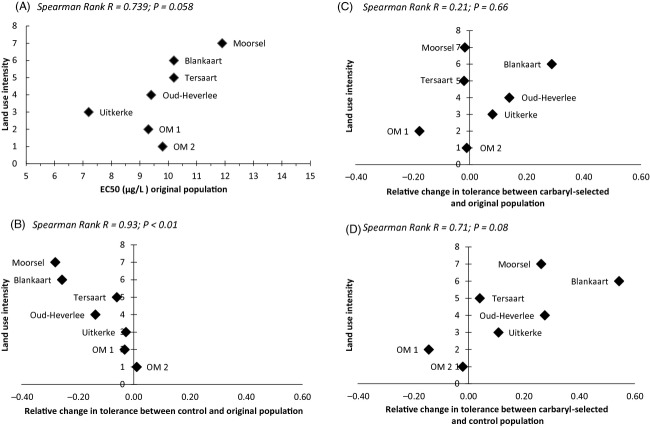
Spearman rank correlations between land use intensity (determined following Coors et al. 2009) and (A) absolute value of the EC_50_ (*μ*g L^−1^) for carbaryl of the original populations, (B, C) the change in EC_50_ during the selection experiment [log_2_ (EC_50_ treatment/EC_50_ original)], and (D) the change in carbaryl tolerance between the control and the carbaryl-selected populations [log_2_ (EC_50_ carbaryl-selected/EC_50_ control)]. Panel B shows that the loss in carbaryl tolerance in control populations compared to the original populations increases with land use intensity around the pond [log_2_ (EC_50_ control/EC_50_ original)]; this correlation is significant. Panel C shows the change in carbaryl tolerance in carbaryl-selected populations compared to the original populations in relation to land use intensity around the pond [log_2_ (EC_50_ carbaryl-selected/EC_50_ original)]; this correlation is not significant. Panel D shows the change in carbaryl tolerance between the control and the carbaryl-selected populations in relation to land use intensity [log_2_ (EC_50_ carbaryl-selected/EC_50_ control)]; this relationship is marginally nonsignificant.

The concentration–response curves of acute exposure to carbaryl for the original field populations and for the experimental populations that were (‘carbaryl-selected’) or were not (‘control’) exposed to carbaryl in our selection experiment are shown in Fig.1 (i.e. carbaryl-selected and control experimental populations of each of the seven original field populations; see also Table S1 for summary statistics). There is a significant main effect of acute carbaryl concentration, population and selection history as well as a carbaryl × population and a population × selection history interaction effect on mortality in the overall analysis (Table1A). Carbaryl concentration is also significant in each of the three comparisons tested (control versus original; carbaryl-selected versus original; carbaryl-selected versus control; Table1B). The population from which the dormant egg bank was derived also significantly shaped the response to carbaryl in all three comparisons (Table1B), illustrating that the populations indeed differ in their concentration–response curves. Selection history (original – control – carbaryl-selected) had a significant effect on the response to carbaryl in the comparison of the control treatment with the original population as well as in the comparison of the control with the carbaryl-selected populations, but not in the comparison of the carbaryl-selected with the original populations (Table1B). Control populations showed less carbaryl tolerance compared to carbaryl-selected populations and compared to the original populations (Fig.1). There is a significant interaction effect between population and selection history in the comparison between carbaryl-selected and control populations (Table1B). This implies that the populations reacted differently to the selection pressures imposed during the selection experiment. This is clearly illustrated in Fig.2D, which shows that while most populations have a higher tolerance to carbaryl in the carbaryl-selected compared to the control selection treatment, one population (OM 1) had lower tolerance in the carbaryl-selected than in the control population. The anova on the EC_50_ values calculated from concentration–response curves estimated for each clonal isolate of the different treatments and populations separately, yielded results in line with the GLiM analysis, with significant main effects of population (reflecting genetic differences among the populations) and selection history (reflecting evolutionary potential). This analysis did not reveal, however, a population × selection history interaction effect (see Table S3).

**Table 1:** Overview of the remaining GLiM models following the stepwise backward elimination process of nonsignificant terms (*α *= 0.05) testing for the effect of carbaryl, population, selection history and their interactions on mortality of *D. magna* in standardized acute toxicity experiments. (A) Results of general model analysing the data of all population × selection treatments; (B) Results of targeted comparisons between selection histories: control versus original, carbaryl-selected versus original, and carbaryl-selected versus control. The two populations obtained in the selection experiment (control and carbaryl-selected population) were compared with each other, and each of them was also compared with the original population in a paired analysis. ‘Carbaryl’ refers to different carbaryl concentration levels in the acute toxicity tests, ‘population’ refers to the pond of origin of the populations, and ‘selection history’ refers to the selection history of the populations (original = prior to selection experiment; control = experimental selection in the absence of carbaryl; carbaryl-selected = experimental selection in the presence of carbaryl)

	df	Residual df	Residual deviance	*F*	*P*
(A)					
All clonal lineages					
NULL		775	15634.1		
Carbaryl	1	774	4976.3	1612.0	<0.0001
Population	6	768	4467.8	12.8	<0.0001
Selection history	2	766	4345.5	9.2	0.0001
Carbaryl × Population	6	760	4242.4	2.6	0.017
Population × selection history	12	748	3985.4	3.2	<0.0001
(B)					
Control population versus original population					
NULL		494	10017.6		
Carbaryl	1	493	2916.8	970.3	<0.001
Population	6	487	2616.3	6.8	<0.001
Selection history	1	486	2541.4	10.2	<0.01
Carbaryl-selected versus original population					
NULL		530	10646.9		
Carbaryl	1	529	3509	990.5	<0.001
Population	6	523	2931.5	13.5	<0.001
Carbaryl-selected versus control population					
NULL		525	10556.8		
Carbaryl	1	524	3470.2	1173.4	<0.001
Population	6	518	3181.4	8	<0.001
Selection history	1	517	3069.6	18.54	<0.001
Carbaryl × Population	6	511	2958.5	3.1	<0.01
Population × selection history	6	505	2742.6	6	<0.001

### Relationship with land use

Similar to the results presented by Coors et al. (2009), the Spearman rank correlation between land use intensity and carbaryl tolerance (represented by the EC_50_) in the original populations shows a marginally nonsignificant positive association (*r* = 0.739; *P* = 0.058; Fig.2A). There is a significant negative correlation between the degree to which populations became less tolerant to carbaryl in the control treatment compared to the original populations and land use intensity in the vicinity of the ponds from which the original populations were obtained (*r *= −0.93; *P* < 0.01; Fig.2B). There is no association between EC_50_ ratio of carbaryl-selected over original populations and land use intensity in the vicinity of the ponds (*r* = 0.21; *P* = 0.66; Fig.2C). There is a tendency for a positive correlation (*r* = 0.71, marginally nonsignificant: *P* = 0.08) between the difference in carbaryl tolerance between the carbaryl-selected and the control treatment populations and land use intensity (Fig.2D).

## Discussion

Overall, our results show that natural populations of the water flea *D. magna* isolated from a gradient in land use intensity differ in their sensitivity to the pesticide carbaryl (cf. significant effect of population in Table1), show evolutionary potential to respond to changes in carbaryl exposure (cf. significant effect of selection history in Table1, our first hypothesis) and differ in the magnitude of this evolutionary potential (cf. significant effect of population × selection history in Table1, our second hypothesis 2, Fig.2).

### Evolutionary potential for carbaryl tolerance

The overall GLiM analysis indicates significant main effects of carbaryl, population and selection history as well as significant population × selection history and carbaryl × selection history interaction effects (Table1A). The significant effect of carbaryl indicates that carbaryl exposure impacted mortality, while the carbaryl × population interaction effect reflects that the slope of the concentration–response curves differs among populations. The significant effect of selection history reveals that the selection experiment impacted carbaryl tolerance of the resulting populations, while the selection history × population interaction effect shows that this effect is dependent on the population. In all three targeted contrasts (Table1B) between different selection histories, carbaryl and population significantly affected mortality. The comparison between the carbaryl-selected and the control populations directly tests for differential evolution in our containers due to carbaryl exposure. In this comparison, the population × selection history interaction is significant, implying that the difference in response to the imposed selection regimes depends on the population of origin. While most populations had a higher EC_50_ value in the carbaryl-selected than in the control populations, one population (OM 1) showed a lower EC_50_ value in the carbaryl-selected than in the control population. Overall, our findings that natural populations can genetically differ in tolerance to pollutants and can show evolutionary potential to adapt to pollutants is in line with the results of several earlier studies that used pesticides (e.g. Tanaka and Tatsuta 2013) or other pollutants (e.g. Muyssen et al. 2005; Agra et al. 2010), and involved *Daphnia* or other organisms (Carriere et al. 1994; Goussen et al. 2013) as model systems.

### Relaxation versus enhancement of tolerance

The comparison of carbaryl tolerance between the control and the original populations showed a reduction in carbaryl tolerance in the control populations. Carbaryl tolerance thus rapidly (within 5–8 generations) decreases in the absence of carbaryl. It is indeed striking that the genetically determined reduction in carbaryl tolerance following a release in exposure to carbaryl was stronger than the genetically determined increase in carbaryl tolerance when exposed to pulses of a high concentration of the pesticide in the selection experiment. Selection history (i.e. treatment during the selection experiment) had a highly significant effect on carbaryl tolerance in the control treatment of our selection experiment, whereas exposure to carbaryl pulses did not result in an overall increase in carbaryl tolerance, even though the concentrations we applied in our selection experiment were quite high. We exposed our experimental populations to three pulses of 32 *μ*g carbaryl L^−1^, while the EC_50_ value in the original populations for neonate survival when exposed to carbaryl has been estimated to be between 6 and 13 *μ*g L^−1^ (present study, Coors et al. 2009). While such high exposure levels in the carbaryl treatment must have strongly impacted mortality in juveniles, the populations persisted. The absence of an evolutionary response upon exposure to the standardized and high concentration carbaryl pulses during the selection experiment may either reflect that there is no evolutionary potential to further increase tolerance compared to that in the original populations or that the selection pressure we imposed was not significantly different from that experienced in the ponds of origin.

Rapid loss of tolerance to pollutants has been observed before. For instance, Levinton et al. (2003) showed a reduction in Cd resistance after 9–18 generations in the oligochaete *Limnodrilus hoffmeisteri* upon restoration of a heavily metal-polluted site. The rapid decrease in pollutant tolerance in the absence of pollutant exposure is generally viewed as an indication that the tolerance has a cost (Sibly and Calow 1989; Van Straalen and Timmermans 2002; Medina et al. 2007; Saro et al. 2012). A cost of tolerance may be related to an increase in energy demand associated with the mechanism to cope with the pollutant (Sibly and Calow 1989; Agra et al. 2011). In earlier work using controlled laboratory experiments, we have shown that carbaryl tolerance has a cost in terms of disease development upon exposure of the *Daphnia* to the endoparasitic bacterium *Pasteuria ramosa*. While our results are very suggestive of an overall cost of carbaryl tolerance acting across populations, our data do not allow us to identify the mechanism of this cost.

### Relationship with land use

Our observation that the tolerance to carbaryl of the original populations tends to increase with land use intensity in the immediate neighbourhood of the habitats they were isolated from (marginally nonsignificant) is in line with previous results obtained with a different set of clones from the very same populations (Coors et al. 2009). As carbaryl concentrations in the field are difficult to quantify, we used land use intensity as a proxy of exposure. Even though the amount of arable land is a crude measure for the use of insecticides, our results combined with those of Coors et al. (2009) are suggestive of a relationship. It could be that the pattern reflects adaptation to the use of acetylcholinesterase-inhibiting insecticides as a class rather than just carbaryl.

The reduction in carbaryl tolerance we observe in our control treatment is significantly associated with land use intensity around the habitats the populations were isolated from. Overall, the patterns shown in Fig.2B,D suggest that populations exposed to intensive land use in the vicinity of their habitat show the highest potential to exhibit an evolutionary response to the experimental treatments. This is in line with expectations, as populations that are more strongly selected for carbaryl tolerance can be expected to show the strongest response upon release from this stressor.

### Methodological considerations

While our study involved an analysis of concentration–response curves of not less than 102 *D. magna* clones, the concentration–response curves of each of the populations are based on an analysis of only 3–6 randomly picked out clones per original and experimental population. The maximum of six clones was set for the sake of feasibility, while the lower number in a number of populations resulted from the fact that several clonal lineages were lost during the relatively long period (> 82 generations) of culture under stock conditions prior to the experiment. While a selection of 3–6 clones is at the lower end to obtain a precise estimate of the concentration–response curve of a population, there are several arguments that support the notion that our concentration–response curves capture real patterns. First, our results relating EC_50_ values of the original populations to land use intensity are nearly identical to those obtained by Coors et al. (2009) using a different set of clones and a slightly larger set of populations (the seven populations studied here are a subset of the ten populations studied by Coors et al. 2009). Secondly, in our analysis of the changes in carbaryl tolerance between the control and original populations in relation to land use, the significant and positive association we observed is very unlikely to originate by chance and reflects that the power of our analysis must have been sufficient to capture the essence of interpopulational differences. Indeed, the key conclusions of our study are based on the general patterns across populations (stronger response in the absence than in the presence of selection by carbaryl, relationship with land use), where less precise estimates of responses of specific populations result in some noise but are not detrimental as long as the overall patterns are strong. In these analyses across populations, our database indeed involves seven times 3–6 clones. In brief, while we want to be careful in our interpretation of the details of the concentration–response curves obtained for each population separately, we feel confident in interpreting the patterns across populations, which was the key aim and approach of this study.

Our observation of an absence of a genetic increase in carbaryl tolerance upon exposure to high doses of carbaryl in our selection experiment is in contrast with previous results, where we provided evidence for the evolution of increased carbaryl tolerance in a standardized 21-day life history test with acute exposure to carbaryl during the first 4 days (Jansen et al. 2011). There are two possible explanations for this discrepancy. First, in our previous study (Jansen et al. 2011), we quantified tolerance using a pulsed exposure test during the first 4 days, while in the present study we carried out static acute toxicity tests. These two approaches can indeed give different outcomes (Naddy and Klaine 2001; Angel et al. 2010). Second, the study of Jansen et al. (2011) involved four rather than seven populations and included one population that showed a strong increased tolerance upon selection (Knokke In). This latter population was not included in the current study due to accidental loss of a number of clones. While this discrepancy underscores that there might be a capacity to evolve an increased tolerance to carbaryl in some populations, the main results of our current study remain unchanged, being that there is evolutionary potential to respond to carbaryl selection, that the reduction in carbaryl tolerance upon release from carbaryl exposure is stronger than the increase in carbaryl tolerance when the animals are exposed to carbaryl in the selection experiment, and that the amplitude of this reduction is related to land use intensity.

### Consequences and perspectives

Our observation that the effect of a release from carbaryl exposure under experimental conditions results in a rapid, genetically determined loss of carbaryl tolerance and that this release is associated with land use intensity, has a number of implications and potential applications. First, our results suggest that the intensive agriculture in the Flemish landscape has exerted an important selection pressure on nontarget taxa, resulting in changes in carbaryl tolerance compared to control conditions. While one may conclude that the capacity to genetically adapt allowed the studied *Daphnia* populations to cope with the stress caused by intensive agriculture, there are two aspects that need to be considered to put our results in context. Obviously, we were only able to test *Daphnia* populations that in one way or another have been able to cope with the stress caused by agriculture in their natural environment. The populations that may have failed to do so have gone extinct and are therefore absent from our data set, as we only sampled habitats that harboured active *D. magna* populations. Also, we only tested a single nontarget species, and a relatively common one that is typically associated with eutrophied waters. This species may therefore be less sensitive to disturbance than more specialized taxa. Our results suggest that even this species is impacted in its evolutionary trajectory by the intensive agriculture that prevails in the study region.

Second, our results also indicate that genetic adaptation to pollution is ecologically costly, given the fast loss of carbaryl tolerance upon release from the selection and earlier measures of costs upon exposure to additional stressors such as parasites (Jansen et al. 2011). The overall picture is that the *Daphnia* populations, even though they are nontarget species for pesticide use, are exposed to continuous selection pressure by pesticides in ponds in the vicinity of crops.

Third, our observation that genetic adaptation to insecticide presence is widespread and (partly) reversible, suggests that one should be aware of the selection history of the *Daphnia* clones used to carry out toxicity tests. Several authors have already suggested to use a genetically diverse battery of *Daphnia* clones to better capture the variation of toxicological response (Barata and Soares 2002; Messiaen et al. 2013). We suggest that one could also use the degree of genetic adaptation to pollutants as a bio-assay to assess past exposure to pesticides in natural populations. Monitoring pesticide concentrations in the field is often highly laborious and costly because of the short temporal scales of exposure and the rapid degradation of the residues (e.g. a half-life of only a few days in the case of carbaryl). Often, nontarget populations will only be briefly exposed to the pesticide during or just following spraying, but concentrations may be very high. If genetic adaptation to such pesticide exposure is common, as suggested by this study as well as by some other reports on genetic adaptation to pollution stress that were published in the last decade (Hoffman and Fisher 1994; Medina et al. 2007; Agra et al. 2011), the level of genetic adaptation to pollution in natural populations can be used to capture the degree of pollution at an intermediate temporal scale and in an ecologically relevant way. While such experiments would involve quite an investment, they might be the most direct way to assess effective pollution exposure at the population level. Moreover, methodological adjustments, such as directly determining concentration–response curves on the population of hatchlings rather than on the resulting clonal lineages, can reduce workload considerably. In developing such an approach, however, it will be necessary to conduct further studies to quantify the degree to which maternal effects and bio-accumulation of pollutants influence the results directly obtained from hatchlings of eggs derived from nature.

## Data archiving statement

Data available from the Dryad Digital Repository: http://doi.org/10.5061/dryad.rg638.

## Supporting Information

**Table S1.** Overview of the EC_50_ value (and 95% confidence interval) with their model parameters (β_0_ and β_1_) for each of the 21 experimental populations: original, control and carbaryl-selected populations of each of the seven original populations. **Table S2.** Overview of average values for four different ecological variables measured in the experimental jars in the acute carbaryl toxicity experiment. **Table S3.** Overview of an overarching analysis of variance (anova) on the EC_50_ values estimated from concentration-response curves for each clonal isolate of the different population × treatment combinations separately, with population and selection history as independent variables. **Table S4.** Percentage of agricultural coverage in an area of 50 m surrounding the pond for the seven ponds involved in the study: 1 – lowest land use intensity, 7 – highest land use intensity.

## References

[b1] Agra AR, Guilhermino L, Soares AMVM (2010). Genetic costs of tolerance to metals in *Daphnia longispina* populations historically exposed to a copper mine drainage. Environmental Toxicology and Chemistry.

[b2] Agra AR, Soares AMVM, Barata C (2011). Life-history consequences of adaptation to pollution ‘*Daphnia longispina* clones historically exposed to copper’. Ecotoxicology.

[b3] Angel BM, Simpson SL, Jolley DF (2010). Toxicity to *Melita plumulosa* from intermittent and continuous exposures to dissolved copper. Environmental Toxicology and Chemistry.

[b4] Barata BC, Soares AMVM (2002). Determining genetic variability in the distribution of sensitivities to toxic stress among and within field populations of *Daphnia magna*. Environmental Science and Technology.

[b5] Carriere Y, Deland JP, Roff DA, Vincent C (1994). Life-history costs associated with the evolution of insecticide resistance. Proceedings of the Royal Society B-Biological Sciences.

[b6] Cerbin S, Kraak MHS, de Voogt P, Visser PM, Van Donk E (2010). Combined and single effects of pesticide carbaryl and toxic Microcystis aeruginosa on the life history of *Daphnia pulicaria*. Hydrobiologia.

[b7] Colbourne JK, Pfrender ME, Gilbert D, Thomas WK, Tucker A, Oakley TH, Tokishita S (2011). The ecoresponsive genome of *Daphnia pulex*. Science.

[b8] Coors A, Vanoverbeke J, De Bie T, De Meester L (2009). Land use, genetic diversity and toxicant tolerance in natural populations of *Daphnia magna*. Aquatic Toxicology.

[b9] Crawley MJ (2007). The R Book.

[b10] De Meester L, De Jager H (1993). Hatching of *Daphnia* sexual eggs: 1: intraspecific differences in the hatching responses of *Daphnia magna* eggs. Freshwater Biology.

[b11] Decaestecker E, Gaba S, Raeymaekers JAM, Stoks R, Van Kerckhoven L, Ebert D, De Meester L (2007). Host-parasite ‘Red Queen’ dynamics archived in pond sediment. Nature.

[b12] Declerck S, De Bie T, Ercken D, Hampel H, Schrijvers S, Van Wichelen J, Gillard V (2006). Ecological characteristic's of small farmland ponds: associations with land use practices at multiple spatial scales. Biological Conservation.

[b13] Goussen B, Parisot F, Beaudouin R, Dutilleul M, Buisset-Goussen A, Pery ARR, Bonzom JM (2013). Consequences of a multi-generation exposure to uranium *on Caenorhabditis elegans* life parameters and sensitivity. Ecotoxicology.

[b14] Hebert PDN (1978). Population biology of *Daphnia**Crustacea, Daphnidae*. Biological Reviews of the Cambridge Philosophical Society.

[b15] Hoffman ER, Fisher SW (1994). Comparison of a field and laboratory-derived population of *Chironomus riparius* (Diptera: Chironomidae): biochemical and fitness evidence for population divergence. Journal of Economic Entomology.

[b16] Jansen M, Stoks R, Coors A, van Doorslaer W, de Meester L (2011). Collateral damage: rapid exposure-induced evolution of pesticide resistance leads to increased susceptibility to parasites. Evolution.

[b17] Kerr DR, Meador JP (1996). Modeling dose response using generalized linear models. Environmental Toxicology and Chemistry.

[b18] Klüttgen B, Dülmer U, Engels M, Ratte HT (1994). ADaM, an artificial freshwater for the culture of zooplankton. Water Research.

[b19] Lampert W (2006). *Daphnia*: model herbivore, predator, prey. Polish Journal of Ecology.

[b20] Levinton JS, Suatoni E, Wallace W, Junkins R, Kelaher B, Allen BJ (2003). Rapid loss of genetically based resistance to metals after the cleanup of a superfund site. Proceedings of the National Academy of Sciences of the United States of America.

[b21] McCullagh P, Nelder J (1989). Generalized Linear Models.

[b22] Medina MH, Correa JA, Barata C (2007). Micro-evolution due to pollution: possible consequences for ecosystem responses to toxic stress. Chemosphere.

[b23] Messiaen M, Janssen CR, De Meester L, De schamphelaere KAC (2013). The initial tolerance to sub-lethal Cd exposure is the same among ten naïve pond populations of *Daphnia magna*, but their micro-evolutionary potential to develop resistance is very different. Aquatic Toxicology.

[b24] Miner BE, De Meester L, Pfrender ME, Lampert W, Hairston NG (2012). Linking genes to communities and ecosystems: *Daphnia* as an ecogenomic model. Proceedings of the Royal Society B-Biological Sciences.

[b25] Muyssen BTA, Bossuyt BTA, Janssen CR (2005). Inter- and intra-species variation in acute zinc tolerance of field-collected cladoceran populations. Chemosphere.

[b26] Naddy RB, Klaine SJ (2001). Effects of pulse frequency and interval on the toxicity of chlorpyrifos to *Daphnia magna*. Chemosphere.

[b27] OECD (2004). http://www.oecd.org.

[b28] Parker ED, Forbes VE, Nielsen SL, Ritter C, Barata C, Baird DJ, Admiraal W (1999). Stress in ecological systems. Oikos.

[b200] R Development Core Team (2007). http://www.r-project.org/.

[b29] Raymond M, Berticat C, Weill M, Pasteur N, Chevillon C (2001). Insecticide resistance in the mosquito *Culex pipiens*: what have we learned about adaptation?. Genetica.

[b30] Saro L, Lopes I, Nelson M (2012). Testing hypotheses on the resistance to metals by *Daphnia longispina*: differential acclimation endpoints association, and fitness costs. Environmental Toxicology and Chemistry.

[b31] Sibly RM, Calow P (1989). A life-cycle theory of responses to stress. Biological Journal of the Linnean Society.

[b32] Tanaka Y, Tatsuta H (2013). Retrospective estimation of population-level effect of pollutants based on local adaptation and fitness cost of tolerance. Ecotoxicology.

[b33] Van Doorslaer W, Stoks R, Duvivier C, Bednarska A, De Meester L (2009a). Population dynamics determine genetic adaptation to temperature in *Daphnia*. Evolution.

[b34] Van Doorslaer W, Vanoverbeke J, Duvivier C, Rousseaux S, Jansen M, Jansen B, Feuchtmayr H (2009b). Local adaptation to higher temperatures reduces immigration success of genotypes from a warmer region in the water flea *Daphnia*. Global Change Biology.

[b35] Van Straalen NM, Timmermans MJTN (2002). Genetic variation in toxicant-stressed populations: an evaluation of the ‘genetic erosion’ hypothesis. Human and Ecological Risk Assessment.

[b36] VMM, Vlaamse Milieu Maatschappij, Vlaamse Milieu (2010).

[b37] Walker CH, Hopkin SP, Sibly RM, Peakall DB (2006). Principles of Ecotoxicology.

[b38] Waterfield GD, Ziberman D, Gadgil A, Liverman DM (2012). Pest management in food systems: an economic perspective. Annual Review of Environment and Resources.

[b39] Wheeler MW, Park RM, Bailer AJ (2006). Comparing median lethal concentration values using confidence interval overlap or ratio tests. Environmental Toxicology and Chemistry.

