# Supplementary material for: Experimental evolution reveals high insecticide tolerance in Daphnia inhabiting farmland ponds
Source: Evol Appl. 2015 Mar 21;8(5):442–53. doi: 10.1111/eva.12253 (PMC4430768; doi:10.1111/eva.12253)
Supplement: Supplementary file 1 — Table S1. Overview of the EC50 value (and 95% confidence interval) with their model parameters (β0 and β1) for each of the 21 experimental populations: original, control and carbaryl-selected populations of each of the seven original populations. Table S2. Overview of average values for four different ecological variables measured in the experimental jars in the acute carbaryl toxicity experiment. Table S3. Overview of an overarching analysis of variance (anova) on the EC50 values estimated from concentration-response curves for each clonal isolate of the different population × treatment combinations separately, with population and selection history as independent variables. Table S4. Percentage of agricultural coverage in an area of 50 m surrounding the pond for the seven ponds involved in the study: 1 – lowest land use intensity, 7 – highest land use intensity. [file eva0008-0442-sd1.docx]

SUPPLEMENTARY

TABLE S1

Overview of the EC_50_ value (and 95% confidence interval) with their model parameters (β_0_ and β_1_) for each of the 21 experimental populations: original, control and carbaryl-selected populations of each of the seven original populations. The number of clones for which the EC_50_ was determined is also given for each population. Original populations are ranked from low to high land use in the neighbourhood of the original pond (see Table S4).

TABLE S2

Overview of average values for four different ecological variables measured in the experimental jars in the acute carbaryl toxicity experiment.

TABLE S3

Overview of an overarching analysis of variance (ANOVA) on the EC_50_ values estimated from concentration-response curves for each clonal isolate of the different population x treatment combinations separately, with population and selection history as independent variables.

TABLE S4

Percentage of agricultural coverage in an area of 50 m surrounding the pond for the seven ponds involved in the study: 1 – lowest land use intensity, 7 – highest land use intensity. (adapted from Coors et al. 2009). “Other” land use may reflect either more natural area (park, forest; OM1, OM2, Oud-Heverlee) or structures (e.g. roads in Moorsel), not related to agriculture. We use the percentage crop as the main criterion to rank the ponds from low to high land use in their neighborhood, but within the ponds with no arable land we use the presence of gardens to differentiate, because some pesticide use is also common in gardens.

TABLE S1

| **Population** | **Selection** | **clones** | **β_0_** | **Β_1_** | **EC_50_ in µg/l (95% CI)** |
| --- | --- | --- | --- | --- | --- |
| OM 2 | Original | 3 | -8.37 | 8.45 | 9.8 (8.7-11.0) |
| OM 2 | Control | 5 | -5.14 | 5.16 | 9.9 (8.7-11.4) |
| OM 2 | Carbaryl-selected | 6 | -8.05 | 8.18 | 9.7 (8.8-10.6) |
| OM 1 | Original | 4 | -7.48 | 7.72 | 9.3 (8.4-10.3) |
| OM 1 | Control | 5 | -8.90 | 9.34 | 9.0 (8.2-9.8) |
| OM 1 | Carbaryl-selected | 6 | -5.44 | 6.09 | 7.8 (7.1-8.6) |
| Uitkerke | Original | 6 | -6.15 | 7.18 | 7.2 (5.6-9.1) |
| Uitkerke | Control | 4 | -5.27 | 6.21 | 7.0 (6.2-7.9) |
| Uitkerke | Carbaryl-selected | 6 | -5.45 | 6.10 | 7.8 (7.1-8.6) |
| Oud-Heverlee | Original | 5 | -4.81 | 4.95 | 9.4 (7.8-11.2) |
| Oud-Heverlee | Control | 4 | -6.27 | 6.88 | 8.2 (7.5-8.9) |
| Oud-Heverlee | Carbaryl-selected | 5 | -8.13 | 7.87 | 10.8 (9.8-11.8) |
| Tersaart | Original | 6 | -7.44 | 7.38 | 10.2 (9.5-11.0) |
| Tersaart | Control | 4 | -7.38 | 7.51 | 9.6 (8.7-10.6) |
| Tersaart | Carbaryl-selected | 6 | -6.40 | 6.42 | 10.0 (9.0-11.1) |
| Blankaart | Original | 6 | -6.04 | 5.99 | 10.2 (8.8-11.8) |
| Blankaart | Control | 5 | -5.19 | 5.78 | 7.9 (6.8-9.1) |
| Blankaart | Carbaryl-selected | 4 | -4.50 | 3.97 | 13.6 (11.1-17.6) |
| Moorsel | Original | 3 | -9.00 | 8.36 | 11.9 (10.4-13.7) |
| Moorsel | Control | 4 | -6.16 | 6.45 | 9.0 (8.1-10.0) |
| Moorsel | Carbaryl-selected | 5 | -8.78 | 8.21 | 11.7 (9.0-15.6) |

TABLE S2

|  | **pH** | **conductivity (µS cm^-1^)** | **oxygen level (mg l^-1^)** | **temperature (°C)** |
| --- | --- | --- | --- | --- |
| ethanol control | 8.28 ± 0.24 | 1147 ± 118.86 | 9.62 ± 0.19 | 19.65 ± 0.22 |
| ADaM control | 8.31 ± 0.30 | 1225 ± 193.26 | 9.58 ± 0.17 | 19.66 ± 0.21 |
| 4 µg l-1 | 8.37 ± 0.24 | 1189 ± 166.84 | 9.64 ± 0.18 | 19.64 ± 0.20 |
| 25.1 µg l-1 | 8.33 ± 0.15 | 1122 ± 49.04 | 9.66 ± 0.16 | 19.43 ± 0.13 |

TABLE S3

| Effect | **Univariate Tests of Significance for EC_50_(48)** | | | | |
| --- | --- | --- | --- | --- | --- |
|  | \| **SS** \| \| --- \| | \| **Degr. of Freedom** \| \| --- \| | \| **MS** \| \| --- \| | \| **F** \| \| --- \| | \| **p** \| \| --- \| |
| \| Intercept \| \| --- \| | 13209.43 | 1 | 13209.43 | 1529.22 | 0.000 |
| \| population \| \| --- \| | 157.83 | 6 | 26.30 | 3.04 | **0.008** |
| \| selection history \| \| --- \| | 60.95 | 2 | 30.48 | 3.52 | **0.032** |
| \| population x selection history \| \| --- \| | 141.71 | 12 | 11.81 | 1.37 | 0.190 |
| \| Error \| \| --- \| | 1097.03 | 127 | 8.64 |  |  |

TABLE S4

| **pond** | **land use coverage** | **ranking** | |
| --- | --- | --- | --- |
| OM 2 | 57% pasture, 43% other | 1 |  |
| OM 1 | 18% gardens, 82% other | 2 |  |
| Uitkerke | 10% crop, 90% pasture | 3 |  |
| Oud-Heverlee | 20% crop, 80% other | 4 |  |
| Tersaart | 25% crop, 75% pasture | 5 |  |
| Blankaart | 64% crop, 36% pasture | 6 |  |
| Moorsel | 70% crop, 30% other | 7 |  |

**Conflict of interest**

There is no conflict of interest.

**Data**

Data for this study are available at: to be completed after the manuscript is accepted for publication.
